# Special Programme for Research and Training in Tropical Diseases-coordinated Multicountry Study to Determine the Burden and Causes of Residual Malaria Across Different Regions

**DOI:** 10.1093/infdis/jiaa605

**Published:** 2021-04-27

**Authors:** Florence Fouque, Tessa Knox

**Affiliations:** 1 Research for Implementation Unit, Special Programme for Research and Training in Tropical Diseases, Geneva, Switzerland; 2 World Health Organization, Port Vila, Vanuatu

**Keywords:** persistent and residual malaria, *Anopheles* insecticide resistance, outdoor biting, unprotected sleeping

## Abstract

The burden and causes of residual malaria were investigated between 2015 and 2019 through 5 research projects coordinated by the Special Program for Research and Training in Tropical Diseases (TDR), cosponsored by the United Nations Development Programme, UNICEF, the World Bank, the World Health Organization (WHO) and the WHO Global Malaria Programme. The 5 projects included 10 countries in 4 WHO regions: Africa, the Americas, South-East Asia, and the Western Pacific. The countries represented a range of malaria endemicities, from low to high levels of transmission. The main findings of the projects indicate that overall the core malaria vector control tools (long-lasting insecticidal nets [LLIN] and indoor residual spraying) were not deployed in the optimal way and/or not efficient in many settings of the supported projects. Furthermore, vector biting behavior and human activity–associated factors strongly contributed to malaria persistence. Changes in vector species composition and abundance, with an increase in outdoor biting, were also reported. Some of these factors may be an adaptation of the vectors to the deployment of the tools and/or can be linked to other sectors, such as agricultural practices, environmental changes, social factors, and water management. Human behaviors and sleeping habits that included activities and sleeping outside villages in unprotected dwellings were another part of the problem. The evidence collated demonstrates the need for new approaches, such as the multisectoral one and new vector control tools, all adapted to the local contexts and integrated into current malaria programs.

Malaria is a vector-borne disease caused by parasites of the species *Plasmodium*, transmitted by infected mosquitoes belonging to the genus *Anopheles*. The major species of parasites infecting humans are *Plasmodium falciparum* and *Plasmodium vivax*. Although great progress has been achieved in reducing the malaria burden and the number of deaths in the past 20 years, an estimated 228 million malaria cases and 405 000 deaths, in 87 countries were reported in 2018 [1]. Malaria prevention and control by means of vector control tools has been recognized as one of the main drivers of the significant decline in malaria. In 2015, it was estimated that insecticide-treated nets (ITNs) and indoor residual spraying (IRS) had contributed to 68% and 10% respectively of the reduction due to interventions [2]. These interventions are consequently recommended as core vector control interventions against malaria, as expressed here: “Core interventions for malaria vector control are applicable for all populations at risk of malaria in most epidemiological and ecological settings, namely: i) deployment of insecticide-treated nets (ITNs) that are prequalified by WHO, which in many settings are long-lasting insecticidal nets (LLINs); and ii) indoor residual spraying (IRS) with a product prequalified by WHO” [3].

However, it has been recognized that these core interventions are not covering the full spectrum of malaria transmission, and even with full coverage some transmission can persist. Residual malaria transmission is thus defined as follows: “Some residual malaria parasite transmission will occur, even with universal access to and usage of ITNs or in areas with high IRS coverage. Residual transmission occurs as a result of a combination of human and vector behaviours” [3]. Residual malaria transmission thus occurs in settings with full and efficient implementation of the core interventions against vectors fully susceptible to the insecticides used. This residual transmission has been linked to vector and/or human bionomics/behaviors which compromise the contact between the vectors and the protective/control measures, such as early evening and/or outdoor biting of mosquitoes and/or human activity at peak biting times away from protected houses [4]. From a geographic perspective, residual malaria has been reported across numerous transmission settings, with several vector species implicated, such as *Anopheles arabiensis* in Africa [5], *Anopheles dirus* in Asia [6], and *Anopheles albimanus* and *Anopheles darlingi* in the Americas [7]. The World Health Organization (WHO) Global Malaria Programme reviewed this topic in a technical note (WHO/HTM/GMP/MPAC/2014.5) and indicated that there is a strong need for new tools and strategies to address residual malaria, both into low and high transmission areas. The development and optimization of these tools necessitates a clearer understanding of the magnitude of the problem.

A collaboration between the Special Program for Research and Training in Tropical Diseases (TDR), cosponsored by UNDP, UNICEF, the World Bank, WHO, and the WHO Global Malaria Programme was initiated in 2015 to provide information on the magnitude of residual malaria transmission in different situations and settings and to determine (1) the contribution of residual transmission to the overall burden of malaria, and (2) the main specific causes of the residual transmission. An open call for applications to support research projects in all the WHO regions affected by residual malaria was launched, to identify through standardized protocols the main factors driving this transmission, including social behaviors or activities that increase human exposure to mosquito bites, environmental changes, and other factors affecting vector resting behavior, feeding, and species composition. The objectives of the applications were to produce updated data from selected settings of low to high malaria transmission on the magnitude of residual malaria and to produce scientific evidence on the causes of this residual malaria through investigations of entomological, social and environmental determinants. After the call and the transparent selection process through an external ad hoc committee, 5 multicountry research proposals were supported encompassing 10 countries within 4 WHO regions (Africa, the Americas, South-East Asia, and the Western Pacific). The mains findings from the projects are reported below.

## ACTIVITIES

### Project A: Residual Malaria Hot Spots in Peru and Brazil—Setting the Stage for Testing Improved Interventions

The overall objective of this project was to investigate the status of the persisting malaria in the Peruvian and Brazilian Amazon areas, along Mazán River (Loreto Department, Peru) and in Mâncio Lima (Juruá Valley, Brazil). Specific objectives included the following: (1) to compare prevalence/incidence of infections among households with or without bed net use and with or without IRS, (2) to investigate the social and environmental determinants of malaria transmission through a combination of household surveys and satellite imagery, and (3) to determine the vector biology metrics and bionomics according to genetic characterization for the main vector *A. darlingi* to clarify whether changing mosquito behaviors could be due to cryptic species.

The results on the coverage of the core tools showed a different situation in Peru and in Brazil. In the Brazilian study site, 50.7% households had received long-lasting impregnated nets (LLINs) in 2014–2015 (Table 1). The deployment of ITNs thus achieved lower than optimal coverage. Conversely, at the Peruvian study site LLIN coverage was relatively high, with 84.4% households having received an LLIN and 99.3% of them using a bed net. However, an overall 70.5% and 99.0% of the population in the Brazilian and Peruvian sites respectively, reported having slept under any bed net (ITN or untreated) the previous night (Table 1).

**Table 1.** Data on Long-Lasting Insecticidal Net Coverage, Bed Net Use, and Indoor Residual Spraying by Project and Location^a^

Project and Countries	Village and Location	Proportion of Households, %			Year of Report
		LLIN Coverage	Bed Net Use^b^	IRS	
A					
Brazil	Mâncio Lima, Juruá Valley	84.4	99	Occasional	2014–2015
Peru	Mazán River, Loreto Department	50.7	70.5	Occasional	2014–2015
B					
Thailand	Tak Province, Tha Song Yang district	55.7	79.5	71.4	2015–2016
Vietnam	Khanh Hoa province	49	95.6	3.01	2015–2016
C					
Burkina Faso	2 Villages around Bobo-Dioulasso	78	NA	NA	2016–2017
Tanzania	Morogoro region	100	94.2	NA	2016–2017
Cameroon	Villages of Olama and Nyabessan	90	89	NA	2016–2017
D					
Ethiopia	Bore Tika (Seka) and Chewaka villages	70	70.4	NA	2016–2017
Kenya	Coastal region in Kilifi County	90	NA	NA	2016–2017
E					
Papua New Guinea	Madang Province (Mugil)	81 to 94	NA	NA	2016–2017
Papua New Guinea	New Ireland Province (Lemakot)	29 to 39	NA	NA	2016–2017

Abbreviations: IRS, indoor residual spraying; ITN, insecticide-treated net; LLIN, long-lasting insecticidal net.

^a^Data were extracted from the research projects on residual and persisting malaria supported by the Special Program for Research and Training in Tropical Diseases. They do not represent country data because they are reported from specific locations at the time of the research study, and they were collected using different methods because each project had distinct objectives.

^b^Including ITNs and untreated nets.

The malaria transmission patterns were also very different in the 2 countries. In the study site in Brazil, the annual parasite incidence was 17.26 per 1000 persons at risk (Table 2), with the highest incidence among men aged 25–39 years and 85% and 13% of cases due to *P. vivax* and *P. falciparum,* respectively. The main vector was found to be *A. darlingi,* and heightened risk of infection was mostly associated with individual behaviors, such as going to bed indoors after 10 pm which increased risk because of the outdoor biting behavior of the vectors [8]. Conversely, the risks were not associated with climate, waking time, household criteria, and/or the use of a bed net. 

**Table 2.** Annual Parasite Incidence and Entomological Inoculation Rate by Project and Location^**a**^

Project and Countries	Village and Location	API, Number of *Plasmodium sp*. Positive Persons per 1000 Population	EIR, Infectious Bites per Person per Year
A			
Peru	Mazán River, Loreto Department	229.57	0–1.75
Brazil	Mâncio Lima, Juruá Valley	17.26	NA
B			
Thailand	Tak Province, Tha Song Yang district	62	NA
Vietnam	Khanh Hoa province	28.3	NA
C			
Burkina Faso	2 Villages around Bobo-Dioulasso	NA	255.45
Tanzania	Morogoro region	NA	8.34
D			
Cameroon	Villages of Olama and Nyabessan	417.4	NA
Ethiopia	Bore Tika (Seka) and Chewaka villages	NA	NA
Kenya	Coastal region in Kilifi County	401	10.95–65.70
E			
Papua New Guinea	Madang Province (Mugil)	279	60.4
Papua New Guinea	New Ireland Province (Lemakot)	49	0–5.37

Abbreviations: API, annual parasite incidence; EIR, entomological inoculation rate; NA, not available.

^a^Data were extracted from the research projects on residual and persisting malaria supported by the Special Program for Research and Training in Tropical Diseases. They do not represent country data because they are reported from specific locations at the time of the research study, and they were collected using different methods because each project had distinct objectives.

In the study site in Peru, malaria incidence rate was 23.0% with men having the higher malaria incidence rate (24.4%) and no differences across age groups. The main vector was also *A. darlingi*, but a very low entomological inoculation rate (EIR; 0–1.75 infectious bites per person per year) (Table 2) and a relatively high number of malaria cases suggests little local transmission, with a highly mobile human population becoming infected in other places. Again, bed net use the previous night or spraying insecticide during the past year was not associated with malaria risks. In both locations, malaria transmission persisted with very different coverage of vector control tools, and although limited access to vector control tools (LLINs and IRS) was detected in the study site in Brazil, most transmission was very likely to be happening from outdoor bites [9]. Consequently, the persisting malaria transmission in this region can be attributed to a residual malaria situation. 

This project also tested new technologies through drone-based high-resolution mapping to identify and characterize *A. darlingi* aquatic habitats [9] and through use of satellite imagery to map malaria clusters in the villages. The clusters were found to be very heterogenous within the same village, with some having high transmission incidence and others lower incidence and when coupled to the mapping of aquatic habitats, this heterogeneity was more linked to mosquito habitats. Finally, the analysis of the genetic background of *A. darlingi* populations showed different degrees of diversity within different locations, but the results did not provide evidence for the occurrence of subspecies and/or genetic bases for behavioral differences.

This study addressing malaria transmission in study sites of 2 countries of the Amazon region shows that with the main vector species, *A. darlingi*, no significant association was found between malaria infection and sleeping under an LLIN, linked to the evidence that the vector is biting mainly outdoors. New technologies were also tested to better map and understand the relationships between disease transmission and the ecology of the vectors. Evidence indicates that this region is most likely facing a residual malaria situation owing to mosquito biting behavior and human behavior involving staying outdoors late into the night. To control this transmission, new tools are needed.

### Project B: Residual Malaria Transmission in the Greater Mekong Subregion—Studies to Examine Magnitude and Identify Causes

The objectives of the project were to determine the magnitude of the residual malaria transmission relative to the overall burden of the disease in the Greater Mekong Subregion, to produce scientific evidence on the entomological, epidemiological, social, and environmental risk determinants, and to propose protocols and tools that can be used to address the residual malaria transmission challenges.

The main findings show that universal coverage of LLINs has not been achieved at the community level for both sites, because only 55.74% of households owned ≥1 LLIN per 2 people in the study site in Thailand, and only 49.03% of households owned ≥1 LLIN in the study site in Vietnam (Table 1). However, it was reported that use of a bed net the previous night was very high, although the type of net was unknown (Table 1). In the study site in Thailand, the malaria incidence decreased in 2016 and prevalence was very low, with mainly *P. vivax* infection (Table 2). Two-thirds of the cases were classified as local transmission, although there is a lot of cross-border exchange within the surveyed area. In the study site in Vietnam, in contrast, the malaria incidence in 2016 was slightly higher than in 2015 (Table 2), with very marked monthly peaks in July and December. The prevalence was also low, but *P. falciparum* was dominant in >80% of the infections. 

In both sites, although there are good practices of seeking healthcare when having fever, there is a low perception of malaria risk, and mosquitoes are not considered as a major nuisance. Over 25% and 70% of people, respectively, stayed overnight on the farm plot with about one-third to 50% of them never or rarely using a bed net there. Although both men and women go foraging in the forest, only men stay overnight in the forest and do not use nets there. A high percentage of households had nets that were observed to have holes or tears, decreasing their protective effect.

These data clearly demonstrate that the full coverage of the at-risk populations in both study sites in the 2 countries was not achieved, because of a lack of nets, lack of replacement of deteriorated nets, or human behaviors in the farm plots or in forest foraging activities. The primary biting vectors in the study site in Thailand are active in the early evening or late morning, with between 20% and 38% of bites occurring when people are not under the nets [10]. High variation in biting rates between villages and different ecological setting was estimated, with variation also in the abundance of primary and secondary vectors, all belonging to 6 species complexes: *A. dirus* sensu lato (s.l.), *Anopheles maculatus* s.l., *Anopheles minimus* s.l., *Anopheles annularis* s.l., *Anopheles barbirostris* s.l., and *Anopheles culicifacies* s.l. This abundance of different vectors with different biting behaviors increases the exposure of the human populations to bites at different times and places when people are not under nets. 

In the Vietnamese study site, 100% of biting from secondary vectors occurred before 10 pm and minimal evidence of transmission risk in the village was found. with almost no vectors collected and a biting rate of less than 1(bite) per night. However, biting in the farm huts and forest occurred at much higher rates, with most bites due to *A. dirus* s.l. (about 95%) or *A. maculatus* s.l. Indoor (farm hut) and outdoor biting rates were 4.38 and 6.21, respectively, and the biting rate in the forest was 4.73. *A. dirus* s.l. biting rates peaked in the evening between 8 and 10 pm, and almost all bites (>90%) occurred before 11 pm [11].

This study reached the conclusion that the current malaria transmission in the study sites of the Greater Mekong Subregion may in part be because full coverage of the core tools has not been achieved. However, even if the coverage were high, about one-third of the transmission is happening when people cannot be under nets, for different reasons. The key gaps that need to be addressed to reduce the current transmission in this region include achieving universal coverage and full efficacy of the bed nets (including in farm huts and forest) and addressing outdoor transmission in the daytime and early evening through novel personal protection tools.

### Project C: Investigating the Magnitude and Drivers of Persistent *Plasmodium* Infections in East Africa (Tanzania) and West Africa (Burkina Faso)

The main objective of the study was to quantify and characterize the residual malaria transmission in communities where transmission persists though LLIN coverage is high (Table 1), through investigations on the biting behavior of vectors, human activities, and environmental factors. The levels of insecticide resistance were also determined.

This project was carried out in 9 villages of the Morogoro region in Tanzania and in 2 villages around Bobo-Dioulasso in Burkina Faso. The main findings show that the EIR in the studied villages in Burkina Faso, with 255.45 infectious bites per person per year, is >30 times higher than in the studied villages in Tanzania, with 8.34 infectious bites per person per year (Table 2). The 2 settings thus present very different transmission patterns. The density of the malaria vectors collected indoors compared with outdoors was varied according to the method of collection but showed more outdoor biting when the human-baited double-net devices were used. The parity rates of the vectors, a good measure of the age of the mosquito population and thus of potential infectivity, were higher in the studied villages in Burkina Faso and higher for *Anopheles funestus* than for *A. arabiensis* in the studied villages in Tanzania [12]. Furthermore, though *A. arabiensis* is now the most abundant malaria mosquito in rural southeastern Tanzania, >80% of the ongoing malaria transmission in the studied villages was estimated to be caused by *A. funestus*, which occurs in smaller numbers but has higher infectious rates. 

High resistance of all malaria vectors to the pyrethroids commonly used in LLINs was observed in the studied villages in both Tanzania and Burkina Faso, and this was confirmed by very low mortality rates observed when mosquitoes were exposed to bed nets collected from the households in the 2 settings. The studies of human behaviors demonstrated that community members in both settings tended to spend most of the early night outdoors, only moving inside their houses at around midnight. No major means of protection against mosquito bites were observed being used outdoors. In the studied villages in Burkina Faso, a significant proportion of people in the studied communities were observed to sleep outdoors at night during the dry season, owing to the hot climate. Again, no major protection against mosquito bites was observed being used in such circumstances. In both settings, the beliefs of the communities around malaria were inaccurate in many cases, and mosquitoes were believed to be just one way of acquiring malaria.

This study concluded that malaria transmission in the 2 settings continues despite high coverage of the recommended tools and may be associated with several factors, such as a decrease in susceptibility of the vectors to the insecticides used for LLINs and IRS, outdoor biting, and the human behaviors of staying or sleeping outdoors with no protection against mosquito bites. Consequently, the core recommended tools of LLINs and IRS are increasingly compromised by insecticide resistance of the vectors, but malaria transmission also persists because of the outdoor biting caused by both mosquito and human behaviors. To address these issues, better management of the insecticide resistance is required, and efforts should be made to provide complementary protection for people spending time outdoors.

### Project D: Understanding Residual Transmission for Sustainable Malaria Control and Enhancement of Elimination Efforts in Africa (Kenya, Cameroon, and Ethiopia)

The objectives of the proposal were to characterize outdoor malaria transmission in different epidemiological settings with scaled-up coverage of LLINs/IRS and to compare with indoor transmission, to determine the contribution of various mosquito behaviors and levels of insecticide resistance to the residual and/or persistent malaria transmission, and to investigate human behavioral/occupational factors associated with exposure to mosquito bites.

The results on the coverage of the recommended tools (LLINs and IRS) show that in the selected studied sites in Cameroon and Kenya, the LLIN coverage was close to 90%, and in Ethiopia it was close to 70% (Table 1). The malaria incidences in the studied villages in the 3 countries were very different, varying from 4% to about 25% (Table 2), except in 1 village in Cameroon with an incidence of 65% and 100% of cases in persons <16 years of age. In Cameroon, high vector species diversity was recorded for the 2 studied sites, and a shift in abundance was reported in the forest, where the primary vectors *Anopheles nili* and *Anopheles moucheti* were less abundant than the secondary ones [13]. Some environmental changes were reported, such as construction of roads and dams that may have affected the densities of the different mosquito species. The densities of mosquitoes resting indoors were also found to be very low, the outdoor biting rates were found to be much higher. In the study sites in Kenya, the main malaria vectors, *A. funestus* s.l. and *A. arabiensis* were also found to be exophilic and highly zoophagic. More mosquitoes were collected outdoors than indoors (57% vs 43%, respectively). The EIR for the study area ranged from 10.95 to 65.7 infectious bites per person per year (Table 2). 

In the villages in Ethiopia, the main malaria vectors were found to be *Anopheles gambiae* s.l., *Anopheles coustani* group, *Anopheles pharoensis,* and *Anopheles squamosus*, with high numbers again collected outdoors and from animal shelters and human households with animals. The peak biting activities for the main vectors were between 6 and 10 pm. Regarding the susceptibility to insecticides, a high resistance level to pyrethroids was detected for *A. gambiae* in all studied sites in Cameroon, in accordance with recent reports on the evolution of pyrethroid resistance across the country. In the studied sites in Kenya, the main vectors had variable susceptibility to insecticide products, and resistance to pyrethroids and DDT was reported as well as in the villages in Ethiopia, where mosquito mortality rates after exposure to different insecticides showed that populations of *A. arabiensis* were resistant, with high variability to pyrethroids and DDT. 

In the villages in Cameroon, the communities were found to be aware of malaria and on the use of preventive vector control measures, but the nets were in poor condition with holes and were sometimes destroyed. In the other settings in Kenya and Ethiopia, community awareness of malaria and the use of preventive vector control measures was not reported, but very high net coverage was found in households, and the human activities in the late evening and early morning were not associated with disease transmission. In most settings in the 3 countries, human sleeping hours were not associated with disease transmission, which was significantly related to the use of nets, though in the Ethiopian villages most children <5 years of age were asleep before 8 pm, but adults aged 15–64 years went to bed later, between 10 and 11 pm. These data show that even children were exposed to mosquito bites between 6 and 8 pm, and most adults were exposed to mosquito bites during the full biting peak (between 6 and 10 pm).

In conclusion, and though the use of LLINs by the village populations in the 3 countries was very high, the persistence of malaria transmission in the study sites may have different causes, such as insecticide resistance, outdoor biting, and exposure during peak biting times. Some shift in vector species and behaviors may be related to the deployment of the tools (LLINs and IRS), with secondary vectors more abundant and outdoor early evening biting behavior. These findings highlight the need to ensure that the recommended tools, such as LLINs and IRS, are efficient, with nets that are not deteriorated and management insecticide resistance ,and to search for additional tools to control outdoor early evening malaria transmission.

### Project E: Understanding Human, Parasite, Vector, and Environmental Interactions Driving Residual Malaria Transmission in Papua New Guinea

Papua New Guinea (PNG) has the highest malaria transmission rates in the Western Pacific Region and exhibits a particularly complex malaria epidemiology with 4 *Plasmodium* species, 13 overlapping *Anopheles* vector species, and diverse geographic, ecological, and climatic zones. The overall objective of the project was to better understand the determinants of persisting malaria transmission in selected studied sites in Madang Province (Mugil Health Center catchment area) and New Ireland Province (Lemakot Health Center catchment area) in PNG. More specific objectives were (1) to determine the prevalence and distribution of malaria infection in the villages located in the 2 different provinces, (2) to characterize the local vector population abundance, composition and behavior, and (3) to identify mosquito-human contact patterns and human behaviors.

The main findings revealed that LLIN coverage differed greatly between the 2 areas, and, surprisingly, the malaria incidence was much higher in the villages having the best coverage. LLIN coverage ranged from 81% to 94% and from 29% to 39% in the studied villages in Madang Province and New Ireland Province, respectively (Table 1). In Madang Province, the incidence of monthly clinical malaria cases was found to be 279 per 1000 population (Table 2), and the prevalence of positive rapid diagnostic test results om symptomatic malaria case patients in community cross-sectional surveys was 3.4%, with about the same proportions of *P. falciparum* and *P. vivax* infections. A marked seasonality was reported, with transmission peaking between August and November, and the malaria cases were mostly in children <10 years old, with the peak of Rapid Diagnostic Test (RDT) positive children at age 5 years.

In the villages of New Ireland Province, the incidence of monthly clinical malaria cases was much lower, at 49 per 1000 population (Table 2) and the prevalence of positive rapid diagnostic test results was 1.9%, although this varied substantially from 0% to 6% across the 4 villages surveyed. Again, the proportions of *P. falciparum* and *P. vivax* infections were very similar. In Madang villages, the dominant vectors were *Anopheles farauti* sensu stricto and *Anopheles koliensis*, with indoor and outdoor EIRs of 24.6 and 35.8 bites per person per year, respectively, for a total of 60.4 infectious bites per person per year (Table 2), representing an infectious risk equivalent to those found in higher-transmission settings in African countries. One of the main vectors—*A. koliensis*—was found to be highly anthropophilic, and the others—*A. farauti*, *Anopheles punctulatus,* and *Anopheles longirostris*—were found to be more opportunistic in their host selection. 

In Lemakot, *A. farauti* was the only vector found in surveyed villages, with 63.2% collected outdoors and a peak biting time between 6 and 9 pm. The estimated human biting rates varied from 0 to 3.26 bites per person per night according to the villages and EIRs varied from 0 to 5.37 infectious bites per person per year (Table 2). The insecticide resistance status of anophelines in PNG is regularly monitored by the National Malaria Control Program, and all vector species were found to be fully susceptible to the products used at the time of this study. The data collected on human behaviors showed that about 40% of the community members in the villages were not sleeping at 10 pm, with a slow increase in people going to bed between 6 and 10 pm. For both studied areas, up to 70% of individuals are exposed (ie, not under an LLIN) during the peak vector biting period in their village.

This study shows that the malaria transmission in the studied sites in PNG is very heterogenous, and the evidence indicates that differences are more related to the vector species than to other factors. In the area with the high transmission rates, the coverage and use of LLINs on susceptible vectors was very high, demonstrating that in this specific situation the core tools (LLINs and IRS) are not protecting the population sufficiently. Malaria transmission is persisting because of outdoor biting and exposure during peak biting times (when people are not under a bed net). It is unclear whether the main malaria vectors have adapted their biting behavior to the deployment of the tools with more outdoor biting in the early evening, or whether their behavior has remained unchanged, in which case the core tools are unlikely to be sufficient to eliminate malaria in the country. Again, these findings highlight the need to develop additional tools to control malaria transmission targeting vectors that exhibit outdoor biting behavior in the early evening.

## DISCUSSION

Malaria transmission is persisting in many settings in the world, despite the deployment of effective LLINs and IRS coupled with appropriate and timely case management. The difference between residual malaria when tools are well deployed and efficient, and persisting malaria due to a lack of deployment or efficiency is clearly not easy to determine, because several confounding factors are acting. This observation makes it almost impossible to determine the burden of purely “residual malaria,” owing to gaps in effectiveness of well deployed interventions. The studies outlined above indicate that vector control tools are not implemented well in many settings, and improved implementation is needed.

The efficiency of the tools may also be compromised by the decreased susceptibility of the mosquito vectors to the insecticide used. The impact of this decreased susceptibility on the LLIN and IRS efficacy is not well known in terms of transmission, but there is a need to clarify the situation in each local context. The management of insecticide resistance through regular monitoring and adaptation measures, such as ease of selective pressure and changes in product class [3], is now a minimum requirement to prolong the efficacy of the core malaria vector control tools of LLINs and IRS.

Several other important factors were identified that contribute to the persistence of malaria. At the sites studied in the African countries, Brazil, Peru, Thailand, Vietnam, and PNG, the outdoor biting early in the evening and concomitant with human activities outdoors is not covered by the currently available protective tools. It is difficult to determine whether this behavior is an adaptation of the vectors to the tools or to the human activities, since both are evolving dynamically in their specific contexts. In other settings with good coverage of LLINs, such as in the studied sites in Vietnam, the foci of malaria transmission moved from villages to farm plots and forests, with secondary vectors implicated. Consequently, new approaches are needed to control the vectors in these new transmission places and to protect the populations in these settings. 

In the villages investigated in Tanzania, another mosquito species is emerging as the main vector [12]. In the studied villages in Cameroon, malaria transmission persists despite good implementation of the recommended tools owing to a high diversity of vector species exhibiting different biting behaviors. The emergence of secondary vectors with different biting behaviors has been found in several places. We do not know if these new vectors are emerging because the primary vectors were effectively suppressed or if they are increasing in relative abundance because of changing human behaviors. Nevertheless, they maintain malaria transmission and are less easy to control because of so many different factors related to bionomics (biting places and times, aquatic habitats, and feeding preferences, among other characteristics).

The main outcomes of these studies reiterate the need to integrate into existing malaria programs new vector control tools that are adapted to target local vector behaviors, such as outdoor biting, and that are appropriate for the local human populations, such as those who engage in forest foraging. For that purpose, new approaches must be developed, including multisectoral approaches in which the health sector and other sectors work together to address the linked challenges, such as insecticide resistance related to the insecticide pressure on agricultural pests, or water management (dam construction) creating new aquatic habitats and resulting in changes in vector composition. The implementation of multisectoral approaches to address the challenges of vector-borne diseases prevention and control will be investigated through further TDR case studies into real-life situations and in relation to theoretical knowledge [14].

## Notes

***Acknowledgments.*** We thank John Reeder, director of the Special Program for Research and Training in Tropical Diseases (TDR), and Pedro Alonso, director of the World Health Organization (WHO)/Global Malaria Programme (GMP), for allowing this collaboration and for their continuous support of the project. Our best thanks also go to Abraham Mnzava, former coordinator of the Vector Control Unit in GMP/WHO, for initiating this project, and to Jan Kolaczinski, coordinator of the Vector Control Unit of GMP/WHO, for discussions and advice on this project. Finally, we deeply thank all colleagues from TDR, WHO, and external institutions that have participated and contributed to this project, including the members of the Steering Committee, Moses Bockarie, Pierre Carnevale, Mario-Henry Rodriguez-Lopez, and Yasmin Rubio-Palis.

***Supplement sponsorship***. The supplement is sponsored by TDR, the Special Programme for Research and Training in Tropical Diseases, based at the World Health Organization.

***Potential conflicts of interest.*** Both authors: No reported conflicts. Both authors have submitted the ICMJE Form for Disclosure of Potential Conflicts of Interest. Conflicts that the editors consider relevant to the content of the manuscript have been disclosed.
